# Rural plastic emissions into the largest mountain lake of the Eastern Carpathians

**DOI:** 10.1098/rsos.172396

**Published:** 2018-05-23

**Authors:** Florin-Constantin Mihai

**Affiliations:** Department of Research, Faculty of Geography and Geology, Alexandru Ioan Cuza University of Iasi, Carol I Blvd, Nr. 20 A, 700505, Iasi, Romania

**Keywords:** plastic pollution, waste dumping, PET, rural, waste management, floods

## Abstract

The lack of proper waste collection systems leads to plastic pollution in rivers in proximity to rural communities. This environmental threat is more widespread among mountain communities which are prone to frequent flash floods during the warm season. This paper estimates the amounts of plastic bottles dumped into the Izvoru Muntelui lake by upstream rural communities. The plastic pollution dimension between seasonal floods which affected the Bistrita catchment area during 2005–2012 is examined. The floods dumped over 290 tonnes of plastic bottles into the lake. Various scenarios are tested in order to explain each amount of plastic waste collected by local authorities during sanitation activities. The results show that rural municipalities are responsible for 85.51% of total plastic bottles collected during 2005–2010. The source of plastic pollution is mainly local. The major floods of July 2008 and June 2010 collected most of the plastic bottles scattered across the Bistrita river catchment (56 villages) and dumped them into the lake. These comparisons validate the proposed method as a reliable tool in the assessment process of river plastic pollution, which may also be applied in other geographical areas. Tourism and leisure activities are also found to be responsible for plastic pollution in the study area. A new regional integrated waste management system should improve the waste collection services across rural municipalities at the county level when it is fully operational. This paper demonstrates that rural communities are significant contributors of plastics into water bodies.

## 1. Introduction

Plastic pollution is a key environmental issue which affects worldwide communities via the global network of rivers, lakes, seas and oceans. As of 2015, approximately 6300 million tonnes of plastic waste had been generated and 79% of it accumulated in landfills or the natural environment [1]. This seems to be a major future environmental crisis, considering that the global waste generation is expected to increase throughout this century to over 11 million tonnes day^−1^ [2]. Plastic debris in the oceans and seas is a notorious environmental threat across the world [3,4]. Marine debris is measured in various geographical areas such as Hawaii [5], the Atlantic Ocean [6], the North Sea [7], the Arabian Gulf [8] and the Adriatic and Ionian Seas [9]. Asian countries are found to contribute the most to plastic marine debris while coastal European Union countries (23 in total) rank eighteenth on the top 20 list of polluters [10]. A new study estimates that reducing plastic loads by 50% in the 10 top-ranked rivers would reduce the total river-based load to the sea by 45% [11]. Lebreton *et al*. [12] calculate that 1.15 and 2.41 million tonnes of plastic waste currently enter the ocean every year from rivers. Mismanagement of plastic waste for instance by littering or landfill of waste constitutes a significant land-based pollution source for the marine environment and no geographical area should be neglected in this regard [13]. Floods may play an important role in river litter issues, cleaning the whole catchment area across upstream localities [14]. These natural hazards may also affect the urban landfills that are improperly located and leaking pollution into water bodies [15,16]. Assessment of plastic waste in rivers has become an emerging field during recent years; the focus has been on urban areas [17,18], but plastic waste generated by rural communities has not been highlighted to date. Development of sound waste management systems is imperative across developing countries [19]. Rural areas could be an important contributor to plastic waste because 1.9 billion people lack waste collection services in rural areas and the coverage rate of the rural population is under 50% in 105 countries [20]. New EU countries are facing the transition from a traditional to sustainable waste management approach following the waste hierarchy paradigm [21]. The separate collection of dry recyclables is a key factor in order to comply with the EU objectives in this sector. Plastic waste, such as bottles made of polyethylene terephthalate (PET), is the main driver in recycling programmes due to increased amounts of this waste in recent decades across the world [22]. Various technical solutions are provided in order to support such recycling and waste recovery programmes [23,24]. National programmes need to support the sustainable local waste management systems, including urban and rural localities. The illegal dumping of waste is still a current threat in Romanian rural regions due to improper waste management services. Development of this sector is emerging under EU regulations, but mixed waste collection and landfill of waste have prevailed in the local waste management options. The rural population is not fully covered by waste management services and separate collection is at an early stage. These conditions allow for plastic pollution from households to pollute surroundings such as local rivers and creeks. Plastic waste that floats is easily carried by flash floods during the warm season into downstream lakes. This paper proposes a novel approach in the assessment of plastic bottle pollution (PET fraction) adapted for the regional scale where geographical features combined with regional waste management data (rural population, the proximity of built-up areas to water bodies, rural waste collection coverage, collection efficiency, and rural waste generation rates) provide an accurate estimation of rural plastic emissions. Izvoru Muntelui lake located in Neamt county, in the North-East Region of Romania, is selected as a relevant case study for this purpose. The dam area acts as the largest waste storage site across the Eastern Carpathians. The data estimated are compared with those collected through sanitation activities carried out around the Izvoru Muntelui lake and its tributaries. This method can be applied to other geographical areas across the globe using either regional waste-specific data (preferably) or by applying reasonable scenarios in cases of limited data. This paper demonstrates that rural localities are significant contributors of plastic emissions into water bodies.

## 2. Material and methods

### 2.1. Study area

**Figure 1.**
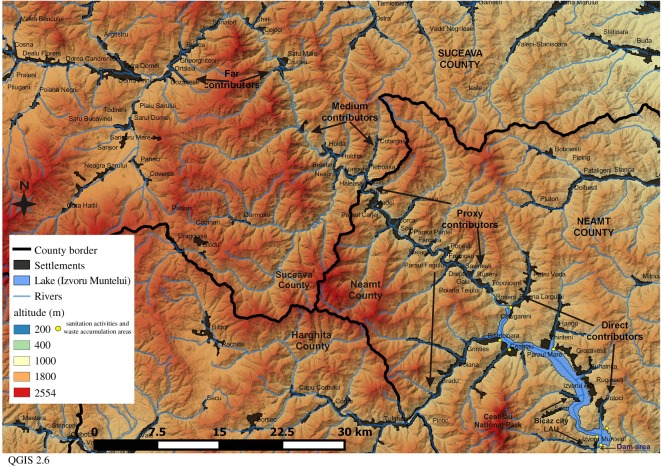
The plastic pollution contributors to the Izvoru Muntelui lake.

Izvoru Muntelui or Bicaz lake is the largest artificial lake in Romania; it is located in the Eastern Carpathians on the Bistrita river course which is the longest mountain river (288 km) in the country. The lake has the following features: (a) area: 32.6 km^2^; (b) length: 40 km; (c) volume: 1250 million m^3^, (d) maximum depth: 97 m; (e) width: 0.2–2 km; and (f) shoreline length: 71 km. The dam was built between 1950 and 1960 and is used to generate hydroelectricity at the Bicaz-Stejaru hydro-plant (more information is available at: https://en.wikipedia.org/wiki/Lake_Izvorul_Muntelui, including a three-dimensional digital elevation model of the dam area). The lake is located near Bicaz city 30 km from Piatra Neamt, the capital city of Neamt County. The lake is crucial water source to surrounding localities and an important tourist attraction in the county, located close to the Ceahlau Mountains National Park. This study identifies four geographical areas which may contribute to plastic pollution of the Izvoru Muntelui lake with different intensities (as shown in figure 1):

(i) Direct contributors to the lake (DCs): villages located in the proximity of the shoreline or near the small creeks which feed the lake such as Potoci, Izvoru Alb (Bicaz City) Ceahlau and Hangu; and Poiana Teiului (consisting of Poiana Largului, Petru Voda, Calugareni, Roseni, Topoliceni and Poiana Teiului villages);
(ii) Proxy contributors (PCs): Poiana Teiului (Ruseni, Galu and Savinesti villages) Farcasa, Borca and Grinties—within Neamt County;
(iii) Medium contributors (MCs): the villages Hăleasa, Lungeni and Neagra which are part of districts; and six villages which are attached to the administrative-territorial unit of Brosteni town: Cotârgaşi, Dârmoxa, Frasin, Holda, Holdiţa and Pietroasa (the main town is excluded from the analysis); and
(iv) Far away contributors (FCs): Crucea commune (Chiril, Cojoci, Crucea and Satu Mare) and Dorna-Arini commune (Dorna-Arini, Cozăneşti, Gheorghiţeni, Ortoaia, Rusca and Sunători).

Brosteni town, Crucea and Dorna-Arini communes are located in the Bistrita valley in Suceava county. These localities may contribute to plastic pollution during strong floods, but the main contributors are the rural localities of Neamt county located upstream of the dam. Medium and far away contributors are both located in Suceava county.

### 2.2. Quantification of uncontrolled rural household waste disposal

A certain fraction of the total household waste generated and uncollected by waste operators is susceptible to uncontrolled disposal via uncontrolled dumps, river dumping or open burning practices in addition to the reuse, recovery and recycling practices performed at the household level. Local environmental reports from Romanian counties stipulate that most of the biowaste fraction of rural communities be diverted from uncontrolled dumps to home composting and animal feed production. In this context, a factor of 0.7 is used for measuring the biowaste losses via unsound waste management practices as a realistic scenario [25]. Organic waste may represent 90% of the domestic waste generation rate in remote rural communities, which is mostly used to feed animals and for composting activities [26], but in Romania, this fraction varies from 47–70% across development regions [27]. The Polish National waste management plan [28] assumes that 70% of biodegradable waste generated in villages and 15% of such waste in minor towns is used for the purposes of composting, feeding animals and in household furnaces. The amount of uncontrolled waste disposed (*Q*_ud_) must be determined to apply waste factors for treatment and reuse of organic waste (0.7) as animal feed or for home composting (kitchen/food waste) and, on the other hand, the reuse of other waste fractions (plastics, paper/cardboard, wood, glass bottles and metals) in other activities (0.1). Paper/cardboard and wood fractions are generally used as solid fuels in households for heating/cooking, whereas metals are collected by individuals and sold to recycling companies. These three waste fractions account for 13% of the household rural waste composition according to the estimations performed by landfill operators in the North-East Region [29]. The recyclables account for 22.5%, including the plastic and glass fractions. Waste generation rates tend to be much lower in rural areas because, on average, residents are usually poorer, possess fewer store-bought items, and have higher levels of reuse and recycling [30]. In this context, a factor of 0.1 of total recyclables generated for household recovery/reuse practices is a reasonable assumption for rural communities. This indicator is calculated at the village level, which is local administrative level 2 (LAU 2, formerly NUTS5) using the following equation if there are no waste collection services across rural municipalities:

2.1 $$Q_{\mathrm{ud}}=\{Q_{\mathrm{wu}}-[(Q_{\mathrm{wu}}-0.7\times Q_{\mathrm{bw}})+(Q_{\mathrm{wu}}-0.1\times Q_{r})]\},$$

where *Q*_wu_ denotes the waste uncollected by formal waste management services (waste operators). This indicator is calculated according to the formula

$$Q_{\mathrm{wu}}=\frac{P\times W_{g}\times 365}{1000},$$

where *P* is the population of the village (no waste collection service (WCS) scenario), *W*_g_ is the *per capita* waste generation rate (= 0.3 kg inhab.year^−1^ (population census 2002 data for 2004–2008) and =0.33 kg inhab.day^−1^ (population census 2011 data for 2009–2012)), *Q*_bw_ is the biodegradable fraction of household waste (65.4%) and *Q*_r_ denotes the recyclables (22.5%); the data for these fractions are extracted from amounts of waste uncollected (*Q*_wu_) using the municipal waste composition of Neamt County (source [31] for the period 2009–2012. The *per capita* waste generation rates are in accordance with regional features. For instance, Ciuta *et al*. [27] calculate a waste generation of 0.31 kg inhab.day^−1^ for rural areas of the North-East Region compared to the flat national rate of 0.4 kg inhab.day^−1^. Waste management plans also suggest an annual increase of 0.8% in the waste generation rate. At the commune level, waste generation rates on a *per capita* basis range from 0.01–0.7 kg inhab.day^−1^ across Neamt county, but caution is necessary due to the poor reliability of the data reported by waste operators [32]. The timescale of the analysis includes two periods as follows:

(i) 2004–2008: when poor or no waste collection services are provided in the mountain area and the wastes are improperly disposed of in the proximity of rivers (no WCS scenario). The rural waste composition data used for calculation of the *Q*_wu_ indicator are those from REPA Bacau [29] such as 62% of biodegradable waste and 23.5% recyclable. Such aggregated data (which are based on landfill operators’ estimations) are almost similar to those from experimental studies conducted in urban areas by Ingleziakis *et al*. [31], except for the plastic fraction; and
(ii) 2009–2012: when regular waste collection services are emerging in the study area after the closure of local dump sites (deadline—16 July 2009) according to [33].

For this period, a waste collection efficiency factor (*C*_ef_) is applied to calculate the amounts of waste generated by villages and uncollected by waste management services. According to Wilson *et al*. [34], the waste collection coverage rates, and wastes captured by the formal waste management system, are categorized as low (0–49%), medium (70–89%), medium–high (90–98%) and high (99–100%). These apply to urban areas where waste management facilities are considered to be more developed than in rural areas, particularly in transitioning and developing countries. The waste management service may be available to 100% of the city, but possibly only 80% actually sign up to and use the service [35]. The collection efficiency scenarios are applied for 40% (low) and 70% (medium) in the study area because illegal dumping practices are still visible even though all rural localities are served by waste operators.

### 2.3 Estimation of river dumping

The amounts of uncontrolled disposal of waste reveal the propensity of rural communities towards improper waste disposal practices. River dumping practices are notorious in mountain regions, particularly in the narrow valleys where dumps are frequently located on river banks. This paper aims to calculate the amounts of waste disposed of by rural localities along a riverbed or creek bank (*Q*_wr_) in the proximity of built-up areas. This indicator is weighted based on the average distance of rivers/creeks to the outer limit of the built-up area [32]. The calculations are made according to the following equation:

2.2 $$Q_{\mathrm{wr}}=(Q_{\mathrm{ud}}-Q_{\mathrm{ud}}\times C_{\mathrm{ef}})\times W_{\mathrm{dist}},$$

where *Q*_wr_ is the waste estimated to be illegally disposed of on river banks/into rivers or creeks by a locality (village) and *W*_dist_ is the weighting factor of the river dumping practice according to the average distance between the built-up area of a locality (village) and the river/creek in its proximity.

The *W*_dist_ has the following values: (a) 0.9 (1–199 m), (b) 0.8 (200–399 m), (c) 0.6 (400–599 m), (d) 0.4 (600–799) and (e) 0.2 (800–1000 m); *C*_ef_ is the collection efficiency factor.

Distances are measured using satellite images provided by Google Earth. The average distances are provided in the electronic supplementary material. This equation suggests that a water body (creek, river, lake) is more susceptible to illegal waste dumping the closer it is to the built-up area. Such hypotheses are confirmed by the monitoring and closure procedure of rural dumps performed in Romania (2009–2010) and field observations. Most of the dumps are located in the buffer zone of 1 km, even in extra-mountainous regions without geographical constraints [32]. The same patterns are found in Poland where most of dumping sites counted during 1994–2016 were located up to 300 m from buildings [36]. The *C*_ef_ is null if there is no regular WCS specific to the 2004–2009 period (no *WCS*)*,* but the two scenarios are further taken into consideration for 2009–2012, such as 40% (WCS40) and 70% (WCS70). Despite the fact that these rural localities are covered by a WCS, they have no reliable records of waste statistics. Significant amounts of PET bottles have been collected in the period 2009–2012 during the cleaning of the Izvoru Muntelui lake, which highlights the gaps in current waste collection services. Limitations in the results derives from the lack of reliable waste statistics data for each commune, which imposes a flat collection efficiency factor to all villages of the study area as for *per capita* waste generation rates, despite possible local disparities. Factors used are specifically for rural communities, which may significantly differ in cases of large urban areas.

### 2.4. Determining the plastic bottles ratio (PET fraction)

This paper aims to calculate the amount of PET bottles generated and disposed of in an uncontrolled manner within the hydrographic catchment of the Bistrita river in the proximity of the Izvoru Muntelui lake. The PET factor of *Q*_wr_ is determined based on municipal waste composition. Ingleziakis *et al*. [31] performed experimental studies concerning the municipal waste composition across capital cities of counties within the North-East Region. In the case of Piatra Neamt, the capital city of Neamt county, the share of plastics is 11.2% of the municipal waste composition and the share of PET bottles is 15% of the total plastics. No experimental studies were conducted in rural areas. The regional waste management plan for the North-East Region [29] provided some data on rural municipal waste composition at the NUTS2 scale (based on waste operators’ estimations) where plastics have a share of 6.5%, but the total of recyclables is slightly above (23.5%) that of urban areas detected by experimental studies (22.5%).

There is a major difference between urban and rural areas concerning the plastics ratio. Furthermore, such discrepancies are also revealed by experimental studies where a share of 5.16% of plastics is determined in the case of the Sercaia commune (Brasov County, Centre Region) by Ciuta *et al*. [27].

The study area of this paper includes mainly rural municipalities, therefore the share of 6.5% is considered in the case of the plastic fraction (2004–2012) and 22.5% of the recyclable fraction during 2009–2012. Three scenarios concerning the share of plastic bottles in the total plastics are used in order to calculate the PET factor:

15%—(QPET15) = 0.065 × 0.15 × *Q*_wr_* *= 0.001 × *Q*_wr_ (0.00975)
30%—(QPET30) = 0.065 × 0.3 × Q_wr_ = 0.02 × *Q*_wr_ (0.0195)
50%—(QPET50) = 0.065 × 0.5 × *Q*_wr_ = 0.0325 × *Q*_wr_ (0.0325)

### 2.5. Flood waste—plastic bottles (PET fraction)

The flood wastes (*Q*_fw_) can be determined according to the relation [32]: *Q*_fw_* = Q*_wr_ _tot_ × *A*_P_ where *Q*_fw_ is the amount of waste carried out by floods from several localities, *A_P_* is the accumulation (storage) period of the waste, expressed in the number of days (d) or per year (yr), and *Q*_wr tot_ is the total waste disposed of by the selected localities along a riverbed or/creek bank (in the proximity of the built-up area).

This paper focuses on plastic dumping and the PET bottles derived from the *Q*_wr_ indicator, which is determined by equation (2.2) as suggested in §2.3.

The accumulation period is difficult to quantify in this region, on the one hand, because of the cold season (December–February) when the Bistrita river and its tributaries are usually frozen and, on the other hand, in the upstream sector of the Bistrita, the ice jam phenomenon often leads to stronger floods in late winter. Thus, due to the climatic conditions in the mountainous sector, the accumulation period is frequent during April–September. At the global level, over 74% of plastic emissions into the oceans occur between May and October [12].

On the other hand, the rivers in the mountainous region are more sensitive to rapid increases of water flows than in the sub-Carpathian region, especially in the case of tributaries with narrow valleys. Such water bodies are more susceptible to the river dumping practices because the built-up areas are closer to watercourses. The spring–summer season has the most rainfall, which leads to flash floods in upstream tributaries, collecting the wastes dumped in smaller catchment areas and transporting them into downstream reservoirs. In this context, accumulation periods are shorter than in other geographical regions. Severe storms, which are frequent in the period from May to July, occur across the entire county, but stronger flash floods that affect the large alluvial plains of major rivers are less frequent in the extra-Carpathian region, thus accumulation periods of waste dumping are longer in such cases [32]. Therefore, at this scale, the accumulation periods in the warm season of the mountain region have variations (of the order of several months to a few weeks). The maximum rainfall period is during April–July in the study area.

### 2.6. Testing scenarios

Different scenarios and variables are applied to explain the role of floods in the plastic pollution issue across the rural localities upstream of the dam. The data results are compared with the amounts of PET bottles collected during sanitation activities (near the dam or across the upstream rural localities). Certain scenarios at the local level are tested to understand which of the parameters are more reliable in measuring the amounts of PET bottles collected.

In the case of the Schit creek, a direct tributary of the Izvoru Muntelui lake which crosses the Ceahlau village with the Durau mountain resort, sanitation activities collected 0.27 tonnes of PET bottles in 2009, and 0.45 tonnes in 2010. These wastes were processed by the local waste operator (SC AGMADY SRL), which transported them to the recycling market. Such data are necessary to analyse the impact of a village on the plastic pollution issue (detailed information can be found in the electronic supplementary material).

The estimated results calculated for Ceahlau village are as follows: 0.067 tonnes (QPET15_noWCS_yr), 0.04 tonnes (QPET15_WCS40_yr), 0.02 tonnes (QPET15_WCS70_yr), 1.35 tonnes (QPET30_no WCS_yr), 0.81 tonnes (QPET30_WCS40_yr), 0.4 tonnes (QPET30_WCS70_yr), 2.2 tonnes (QPET50_noWCS_yr), 1.32 tonnes (QPET50_WCS40_yr) and 0.66 tonnes (QPET50_WCS70_yr).

The results of these nine applied scenarios suggest that:

(i) the data of the QPET15 scenario are very low, thus the share of PET bottles is likely to be greater than 15% of the total plastics and therefore QPET15 is eliminated from further analysis;
(ii) Ceahlau village is covered by waste collection services in 2009 and 2010, thus the scenarios with no WCS are excluded; and
(iii) three scenarios out of nine have values close to the PET bottles collected in 2010, such as 0.81, 0.4 and 0.66 tonnes. The estimated amounts of plastics dumped should be larger than those collected during the sanitation activities because such activities are frequently performed during particular periods of the year; not all waste generated is expected to be collected by ecological activities; the tourism and leisure activities contribute supplementary inputs to the plastic pollution issue; and flash floods may transport the plastic bottles far away from original dumping sites. In this case, QPET30_WCS40_yr and QPET50_WCS70 have more reliable results related to 0.45 tonnes of PET bottles collected in 2010. Another test is applied in order to reveal the impact of several villages in the proximity. Some shorter periods must be chosen from the historical records of flood waste sanitation campaigns (2005–2012) to exclude the external contributors (medium and far away contributors).

The Water Management System of Neamt County (SGA Neamt) implemented a cleaning action programme of water bodies and their banks, collecting the PET packaging and other wastes in April 2008. This action was also supported by the National Environmental Guard (County Commissariat), Inspectorate for Emergency Situations (ISU Neamt), SC Hidroelectrica S.A.-S.H. Bistrita, Bicaz City Hall and local authorities of Hangu, Poiana Teiului, Grinties, Ceahlău, Tasca and Alexandru cel Bun. This initiative collected about 1.5 tonnes of PET bottles. After a month, the flood wastes which had been discharged into the Izvoru Muntelui lake necessitated a new sanitation action programme, collecting 0.4 tonnes of plastic bottles. Therefore, the plastic pollution impact of direct and proxy contributors can be accurately assessed using an accumulation period of one month (30 days) between these two sanitation campaigns. The following results using the scenario (QPET30_noWCS_30d_p02_2008) suggest that direct tributaries dumped 0.48 tonnes of PET bottles, or 0.62 tonnes of PET bottles if the proxy contributors are included (37 villages in total). The similar values with the amounts of plastic bottles collected support this method and the scenario used. The results argue, in this case, that only localities in the proximity of the shoreline are responsible for pollution.

Both particular situations highlight that the proposed method is reliable and only some parameters are suitable for further analysis (e.g. QPET30; QPET50; no WCS; WCS40; WCS70). This method allows an assessment of the plastic pollution threat for various periods during a year.

## 3. Results and discussion

### 3.1. Waste management background in the study area

The poor waste management facilities, particularly in rural areas, lead to plastic pollution of water bodies. The dry recyclables are often found in local dumps and their potential recovery is lost. Furthermore, plastic bottles are disposed of on flood plains or riverbanks, polluting the rivers and their tributaries in the proximity of households. Mountain rivers are the most susceptible to waste dumping due to several factors such as: (i) the lack or poor coverage rate of the rural population by regular waste collection services (2003–2009); (ii) the poor efficiency of waste collection services even where these are provided; (iii) the short distances between built-up areas and watercourses; (iv) the lack of separate waste collection systems; (v) poor environmental awareness; and (vi) sanitation fees and the low incomes of inhabitants. Lakes, including artificial lakes and reservoirs, can act as temporary storage facilities for all kinds of litter, but it is the rivers that are the key pathways to lowlands and coastal areas [37]. This study points out the role of a dam lake as the largest waste storage facility in the Eastern Carpathians where large amounts of waste are accumulated from upstream rural localities mainly following warm-season floods. In Neamt county, most of the population lives in rural areas (62.3% in 2010) where solid waste management facilities were poorly developed until EU accession. The expansion of waste collection services towards rural areas emerged after the closure of rural dumps in July 2009 [38]. In the study area, local municipalities are obliged to provide waste collection services or to delegate them to private waste operators. Mixed waste collection prevails in upstream localities of the Izvoru Muntelui lake. This is performed through the door-to-door system using bags or bins, or through collection points (containers—1.1 m^3^). The main waste operator in the study area is SC Agmady SRL Durău, which collected the waste from Ceahlău, Grinties, Hangu, Poiana Teiului and Borca communities in 2011. The waste collected is transported by waste operators to the Targu Neamt city landfill site. Separate collection facilities are poorly developed around Izvoru Muntelui lake, which explains the vulnerability of the study area to plastic pollution.

### 3.2. The role of flash floods in the plastic pollution of the Izvoru Muntelui lake

The formal waste collection services are competing with the unsound waste disposal practices of rural inhabitants (at any time and almost anywhere) in the proximity of human settlements and at no cost. The Bistrita river and its tributaries carried all floating waste disposed of in the local catchment areas towards downstream localities. A database of flood wastes collected from the Izvoru Muntelui lake is computed for the period 2005–2012 based on data provided by *SGA* Neamt, Hidroelectrica Piatra Neamt, National Environmental Guard (County Commissariat) and local municipalities which had initiated several cleaning activities of water bodies across the upstream mountain sector. Plastic bottles (PET fraction) and wood wastes were the main fractions collected during sanitation activities (2005–2012).

The surroundings of the Poiana Teiului viaduct are an important waste accumulation area with the following key contributors: Borca, Farcaşa, Poiana Teiului (including the Bolătău creek). Sanitation activities collected about 60 m^3^ of PET bottles during 20–27 July 2006, approximately 1.56 tonnes with a specific density of 26 kg m^−3^ in refuse sacks according to WRAP [39]. In this particular area, the PET bottles across proxy, medium and far away contributors may accumulate after stronger floods. During the same period, downstream, the cleaning activities performed by the Hangu commune collected 3.1 tonnes of PET bottles and 6 tonnes of other wastes; near the dam, similar actions collected about 0.5 tonnes of plastic bottles during 14–17 July 2006. Therefore, the flood wastes have been accumulated in different areas of the Izvoru Muntelui lake as revealed by the yellow dots on the map (figure 1). Sanitation activities collected about 5.16 tonnes of PET bottles and 6 tonnes of other wastes from 14–27 July 2006.

Data from sanitation activities carried out around the Izvoru Muntelui lake (2005–2007) is compared with the assessment method using population census data of 2002 and the various scenarios are shown in table 1. The amounts of plastic bottles collected (2005–2007) via sanitation activities (10.16 tonnes) in comparison to those estimated above reveal the fact that pollution is mainly local (direct and proxy contributors). Furthermore, only a fraction (15%) of the total plastics dumped and scattered across the catchment area (22.52 × 3= 67.56 tonnes via QPET30_noWCS_yr_t_p02 scenario) had been collected in this period. This situation explains the huge amounts of PET bottles collected during the stronger floods in July 2008 and June 2010.

**Table 1.** Comparative analysis of PET bottle dumping into the Izvoru Muntelui lake between those collected via sanitation activities and the assessment method (2005–2008).

sanitation PET bottles (tonnes)	period	location	assessment method (tonnes)	scenario used	contributors
2.5	June 2005	Izvoru Muntelui lake	22.52	QPET30_noWCS_yr_t_p02	DC + PC (37 villages)
			5.55	QPET30_nowcs_90d_t	DC + PC (37 villages)
5.16	July 2006		6.47	QPET30_nowcs_105d_p02	DC + PC (37 villages)
0.5	(a) July 14–17	near the dam			
1.56	(b) July 20–26	Poiana Teiului area			
3.1	(c) July 17–24	Hangu area			
2.5	April 17–19 2007	near the dam	1.851	QPET30_nowcs_30 d_p02	DC + PC (37 villages)
93	2008	near the dam	22.52	QPET30_noWCS_yr_t_02	DC + PC (37 villages)
total 103.16	2005–2008		22.52 × 4 = 90.08	QPET30_noWCS_yr_t_02	DC + PC (37 villages)

The most susceptible period of flash floods is between April and August. March is generally exposed to floods caused by the ice jam phenomenon (Bistriţa upstream sector with a high frequency in recent years) and melting snow [40]. The database of SGA Neamt confirms that most of the wastes collected across Izvoru Muntelui lake are discharged by flash floods during the June–July period, April at the earliest and no later than September, as illustrated in table 2. Hidroelectrica Bistriţa conducted several sanitation action programmes around Izvoru Muntelui lake during the warm season (April to September 2008). These sanitation activities collected 55.3 tonnes of PET bottles and 1241 tonnes of wood waste, plus 37.7 tonnes by the end of the year, resulting in 93 tonnes of total plastic collected in 2008. Plastic bottles disposed of into local catchment areas by surrounding localities are often carried out by flash floods towards the lake during April–September. In the period 2005–2008, sanitation activities collected approximately 103.16 tonnes of PET bottles of which 93 tonnes were collected in 2008. There is clear evidence how these strong flash floods influence the amounts of plastics discharged into Izvoru Muntelui lake. In five years (2004–2008), about 112.6 tonnes of PET bottles (22.52 tonnes × 5) were estimated as being dumped into the Bistrita river and its tributaries, according to the scenario QPET30_noWCS_yr_t_02 by direct and proxy contributors. In this case, the flash floods of 2008 discharged into the lake 82.59% of PET bottles (93 tonnes) dumped in the catchment area (2004–2008) within Neamt County.

**Table 2.** Flood waste collected (tonnes) in the proximity of the Izvoru Muntelui dam. Data source: SH Bistrita _ Piatra Neamţ. SGA Neamt.

waste collected (tonnes)	2008 (May–Dec) 245 days	2009 (May–Nov) 215 days	2010 (March–Dec) 306 days	2011 (Jan–Oct) 304 days	2012 (May–June) 61 days
PET bottles	93	43.53	128.19	10.6	4.18
household	—	—	—	19.52	1
wood	1241	289.5	1362	314.5	62

Table 2 highlights the fact that the largest amounts of plastics and wood waste were collected during the periods of heavy rainfall with strong flash floods such as those of July 2008 and June 2010. Large amounts of plastic bottles were discharged into the lake via Bistrita river and its upstream tributaries as illustrated in table 1.

Significant amounts of wood are collected from the lake through sanitation campaigns. These occur mainly from non-household sources such as logging activities and flash floods of small catchment areas of Bistrita tributaries. Wood waste, as a fraction of household waste in rural communities, is insignificant (1%) because it is reused as solid fuel [27]. In 2009, 43.53 tonnes of PET bottles were collected, but direct and proxy localities dumped 20.86 tonnes (QPET30_noWCS_yr_t_p11) plus 8.54 tonnes (the rest of PET bottles from 2004–2008), resulting in a total of 29.4 tonnes (67.53% of the total collected). The remaining 14.12 tonnes and the huge amounts of plastics collected in 2010 suggest that PET bottles accumulated in Izvoru Muntelui lake include those generated not just by contributors in close proximity, but also by those from further afield.

The major flash floods (during 2008–2010) had cleaned all PET bottles dumped into the Bistrita river and its tributaries, feeding across 56 villages. The total amount of PET bottles collected during 2005–2010 is 275.32 tonnes. In reality, this amount is likely to be larger, because not all sanitation activities weighted or estimated the amounts of plastics collected and, moreover, the impact of tourism and leisure activities is not assessed. Direct and proxy contributors (37 villages of Neamt county) dumped 137.6 tonnes of PET bottles (50.0% of the total collected) in the period 2004–2010 (275.32 tonnes) as illustrated in table 3. Medium and far away contributors (19 villages of Suceava county) dumped 97.84 tonnes of PET bottles during 2004–2010. The total amount which resulted is 235.44 tonnes, almost 85.51% of total plastic bottles collected during 2004–2010.

**Table 3.** Plastic pollution contributors to the Izvoru Muntelui lake during 2004–2012.

period	assessment method (tonnes)	scenario used	contributors	sanitation (tonnes) (waste collected)
2004–2008	112.6 (22.52 × 5)	QPET30_noWCS_yr_t_p02	DC + PC (37 villages)	103.6
2009–2010	25 (12.521 × 2)	QPET30_wcs40_yr_t	DC + PC (37 villages)	171.72
2004–2008	87.54	QPET30_noWCS_yr_t	MC + FC	
2009–2010	10.3 (5.15 × 2)	QPET30_WCS40_yr_2010	MC + FC	
2004–2010	235.44		57 villages	275.32
2011 (306 days)	10.42	QPET30_WCS40_2011_304d	DC + PC (37 villages)	10.6
May–June 2012 (61 days)	2.09	QPET30_WCS40_2012_61d	DC + PC (37 villages)	4.16
	2.03	QPET30_WCS70_2012_61d	DC + PC (37 villages)	
Mar–June 2012 (122 days)	4.18 (2 × 2.09)	QPET30_WCS40_2012_122d	DC + PC (37 villages	4.16

These results point out that rural localities may not be solely responsible for the waste dumping issue. Tourism and leisure activities, particularly in the summer season, may have a significant impact via illegal waste-disposal practices in the proximity of the shoreline. Mihai [41] revealed that the impact of tourists on the waste generation rate across rural areas could be between 3–5%, taking into account the registered number of tourists provided by the accommodation units. In this context, the number of tourists could be 2–3 times larger in reality, particularly in this region of the county. Therefore, the impact of tourism and leisure activities on the surroundings may be responsible for 10–15% of PET bottles dumped into the lake. The amount of plastic collected significantly decreased in 2011 and 2012 compared to the 2008–2010 period, which outlines the role of strong floods in the pollution issue of the Izvorul Muntelui lake.

This assessment method reveals that, in 2011, about 10.42 tonnes of PET bottles were dumped using 304 days as an accumulation period (QPET30_WCS40_2011_304d). This result is similar to the amounts collected in the same period near the dam (10.6 tonnes). Direct and proxy contributors are responsible for plastic pollution during the previous years (2005–2007) with a surplus generated by the tourism sector. The main waste accumulation areas are the dam, the tourist river port of Potoci and the viaduct of the Poiana Teiului commune.

Sanitation activities supported by SGA Neamt have continued in 2009–2012 on Bolatău, Farcaşa and Schit creeks. In 2012, such activities cleaned about 35 km of hydrographic networks from several watercourses which feed into the Bistrita river (the Izvoru Muntelui lake) such as Bistrita, Pârâul Pantei, Sabasa, Stejaru, Bolatau, Schit, Bistricioara, Hangu and Buhalnita, collecting almost 120 m^3^ of waste (of which 23 m^3^ of PET was collected, equivalent to 0.6 tonnes). The ecological campaign ‘Let's do it Romania' conducted a mini-campaign around the viaduct of Poiana Teiului and collected about 1.5 m^3^ of waste in 2010, 10 m^3^ in 2011, and 3.5 m^3^ in 2012.

In 2012, only 4.18 tonnes of PET bottles were collected during the May–June campaign (61 days). The assessment method estimates 2.09 tonnes of PET bottles dumped by direct and proxy contributors in 61 days (QPET30_WCS40_2012_61d), which corresponds to 50.4% of the total collected. The scenario which uses a *C*_ef_ of 70% (QPET30_WCS70_2012_61d) shows an insignificant difference to be 2.03 tonnes of plastic bottles. The difference between the amounts collected and those estimated are explained by the PET bottles dumped by the same contributors during the March–April period (61 days). Thus, between March and June (122 days) the total amount of PET bottles dumped into the lake is 4.18 tonnes. Similar values between the historical records of sanitation activities and estimation data (table 3 and §2.6) point out that plastic pollution of water bodies can be monitored through the method proposed in this paper.

### 3.3. The impact of plastic pollution

**Figure 2.**
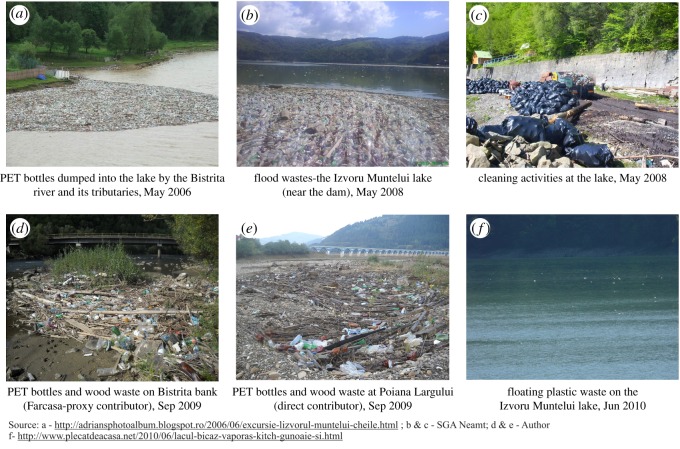
Plastic pollution of Bistrita river and the Izvoru Muntelui lake.

Plastic bottles, particularly the PET fraction, have a short life as products and a long life as post-consumer waste if it is not recycled. In this context, the unmanaged plastic waste poses serious challenges to ecosystems, water bodies and human settlements [42]. Plastic pollution of small rivers in the upper Bistrita catchment area compromises the efficiency of the surface drainage system, amplifying the destructive force of flash floods on the surroundings. Plastics are mixed with other fractions (wood, bulk waste, packaging materials) which accumulate near the local bridges or the river banks, as shown in figure 2.

These areas may increase the material losses during future floods. Plastic pollution resulting from strong floods damages the landscape around the Izvoru Muntelui lake, which is a key tourist area in the region. The large surface of the lake is covered by plastics and woods, which affect the local navigation, fishing and leisure activities. The microplastics of the lake may be a serious threat to the local ecosystem, particularly for animals in search of food (fish, birds). At the global level, the plastic pollution threat is geographically widespread and ecological models predict the risk of plastic ingestion to 186 seabird species, particularly in the southern boundary of the Indian, Pacific and Atlantic Oceans [43]. Lakes are vulnerable to microplastic pollution in the proximity of large industrial areas such as the Great Lakes [44,45] or even in the high mountain lakes of Mongolia [46]. Urban litter is a source of marine pollution via rivers in the coastal areas [47,48]. European surface waters are exposed to microplastic pollution in the Paris region of France [49], the Rhein-Main area of Germany [50], the lakes of Switzerland [51] and the Bolsena and Chiusi lakes in central Italy [52]. Microplastic pollution seems to be a widespread issue of a global scale, but plastic pollution in terms of large fractions such as PET bottles is a serious challenge for those countries with deficiencies in waste management systems across urban and rural areas.

Lu & Wang [53] point out the role of rural solid waste management for a crucial non-point pollution source of Dianchi Lake catchments in China. Illegal dumping of waste plays an important role in the contamination of rivers, coastal waters and shorelines in Chile [54]. Rural areas of Eastern and Southeastern Europe have poor waste management facilities, which leads to river dumping practices [32,55]. Milovanovic [56] reveals that significant loads of pollutant are discharged due to the disposal of solid waste in the irregular landfills in the upper part of the Axios/Vardar River catchment. Romanescu *et al*. [57] examined the sources of pollution of water in the Bistrita catchment focusing on the industrial sector, but the waste management sector was not taken into consideration. Landfills and illegal waste disposal sites (as non-point pollution sources) are also serious threats to public health and river ecosystems. The lack of proper waste management services can heavily pollute freshwater systems with consumer plastics even in sparse areas such as the surroundings of the Hovgol lake in Mongolia [46]. A total of 409 debris items (10.3 kg) were collected during the shoreline surveys, which is almost insignificant compared with those collected in the Izvorul Muntelui lake, but plastic bottles have a ratio of 30% of the total plastic like in the QPET30 scenario used in this study. The Izvorul Muntelui lake is located in the Siret hydrographic district as part of the Danube river basin. During the major floods in Romania, the Danube collects all the impurities (including plastic debris) which may be carried via the delta into the Black Sea. Lechner *et al*. [58] outline that plastic litter is a serious threat to Europe's second largest river. The plastics fraction has the highest share (45.2%) in the marine litter composition found in Constanta Bay (Black Sea), of which 14.5% are bottles [59]; in other words, plastic bottles represent roughly 30% of the total plastic litter in the bay, the same as the QPET30 scenario used in this paper.

Poor or insufficient waste management practices in the Adriatic-Ionian macroregion lead to plastic pollution of beaches including the semi-rural or natural areas. Vlachogianni *et al*. [9] classified these beaches according to their cleanliness (clean coast index) as follows: (i) very dirty semi-rural (Zaglav, Croatia), (ii) dirty semi-rural (Greece, Slovenia) or semi-urban (Montenegro), (iii) moderate, mainly urban and semi-urban types, (iv) clean, mostly semi-urban and (v) very clean, remote/natural (Greece) and semi-rural (Mega Ammos, Greece). River floods, strong persistent winds and summer population peaks along the coast due to the tourism industry mediate the transfer of litter from inland and coastal sources into the northwestern Mediterranean Sea [60]. Such studies must be further developed in various geographical areas to understand and quantify the magnitude of marine and river plastic pollution. Plastic emissions from lands into oceans rely upon the following indicators: (i) waste generation per capita, (ii) the proportion of waste that is plastic and (iii) the percentage of waste that is mismanaged [13]. Global assessments of plastic waste as a land-based source do not reflect the geographical disparities across a country or region in terms of waste management practices. The lack of reliable regional waste statistics data increases the uncertainties related to the magnitude of plastic pollution associated with unsound waste management practices which contribute to plastic pollution of surrounding water bodies and ultimately of seas and oceans. *Per capita* waste generation rates based on World Bank estimations reflect at best a national or capital city average but do not define the regional or urban–rural differences within a country [30], or waste generation rates are assumed based on the national economic development level. The littering behaviour rate of plastic waste is assumed to be 2% across the globe; however, this value is based on a US study [10] in the context of major discrepancies of waste collection and disposal practices across countries and regions. Regional assessment of river and marine plastic pollution must be further developed taking into consideration the regional and local waste management performances. Urban and rural waste generation rates, urban and rural waste collection coverage, and efficiency rates are key items of information to evaluate the illegal waste disposal practices which include open dumping, river or marine dumping, and open burning practices. This should be the starting point to further assess the plastic emission into water bodies at regional and local levels, particularly for countries and regions where waste collection coverage and efficiency is not 100%. This paper proposes such a methodology focusing on rural communities with gaps in the waste collection systems.

### 3.4. Future perspectives in plastic waste management

The EU aims to recycle and recover 50% of the municipal waste fraction by 2020. This target is difficult to achieve in Romania due to poor separate waste collection schemes across rural areas. Plastic pollution is a widespread environmental threat to water bodies in the proximity of rural settlements, particularly in mountainous regions. EU and national regulations impose the separate collection of five recyclable streams (plastics, paper/cardboard, metal, wood and glass) to be diverted from landfills. Source-separated collection schemes of such recyclables need to be further developed across rural municipalities. The new regional integrated waste management system at the county level, financed through the EU structural fund 2007–2013, aims to extend the waste collection coverage towards rural localities, to implement separate collection schemes and a new sanitary landfill site in the Girov commune which should serve all municipalities of Neamt county [38]. The county-integrated waste management system includes three transfer stations (Targu Neamt city, Tasca commune and Cordun commune) and a sorting station (Cordun commune). The rural localities of the study area should benefit from a separate waste collection system where plastics could be disposed of in special bins or containers. The residual municipal waste fraction (without dry recyclables) should be transported to the Targu Neamt transfer station, and subsequently disposed of at the regional sanitary landfill (Girov commune). The post-consumer PET bottles collected from the population, economic agents and local institutions should be sent to recycling units. As an example, the Greentech Buzau is one of the largest companies in southeast Europe involved in the recycling process of PET bottles. It claims to cover 90% of the plastic waste buyer market in Romania. The plastic bottles collected during sanitation activities around the dam area are sold to such companies, preventing their disposal in urban landfills. The recycling companies pay between 0.6 and 1 RON per kg of plastic bottles (1 EUR = 4.6 RON). Such cleaning activities will be needed in the coming years until the regionally integrated waste management system is fully operational. Environmental awareness and correct source-separated waste collection are key points to mitigate the plastic pollution issue and to increase the recycling process of post-consumer PET bottles.

## 4. Conclusion

The magnitude of plastic pollution in the Izvoru Muntelui lake is revealed by the large amounts of plastic bottles (290 tonnes) collected during sanitation activities (2005–2012). This paper examines the role of floods and the geographical dimension of rural plastic pollution in the study area. The source of plastic pollution is mainly local (direct and proxy contributors) during 2005–2007 and 2011–2012. On the other hand, the major floods of July 2008 and June 2010 collected most of the plastic bottles scattered across the Bistrita river catchment (56 villages) and dumped them into the lake. The assessment method outlines that besides rural municipalities (235.5 tonnes of plastic waste disposed, almost 85.51% of total plastic bottles collected through sanitation campaigns during 2005–2010), tourism and leisure activities may also be responsible for plastic pollution which occurred in the last decade in the study area. The method proposed is validated as a reliable tool in the assessment process of river plastic pollution. Such analysis is new to the literature; river dumping practices are still a widespread waste disposal option across the globe. The extension of a new regional integrated waste management system will mitigate this environmental threat in the study area. This paper provides an insight into the regional dimension of plastic pollution (PET bottles) and takes into consideration the rural waste management indicators combined with the geographical proximity of human settlements and water bodies.

Such analysis could help the regional and local decision-makers to adjust their environmental policies and regulations so as to better manage emerging threats and to improve waste management options across rural communities. Also, reasonable scenarios could be applied further in this methodology to assess the potential threat of local water bodies to plastic pollution in countries and regions with limited waste management data, particularly for smaller urban areas and rural communities. Plastics dumping is a serious global environmental threat to oceans, seas and rivers. More attention should be accorded to the river dumping of plastics and this issue should be further studied because rivers are a major land-based source of plastics for the global network of seas and oceans.

## Supplementary Material

ESM_scenarios_ model_village_level

## Data accessibility

The datasets supporting this article have been uploaded as part of the electronic supplementary material.
